# Physical exercise in overweight to obese individuals induces metabolic- and neurotrophic-related structural brain plasticity

**DOI:** 10.3389/fnhum.2015.00372

**Published:** 2015-07-01

**Authors:** Karsten Mueller, Harald E. Möller, Annette Horstmann, Franziska Busse, Jöran Lepsien, Matthias Blüher, Michael Stumvoll, Arno Villringer, Burkhard Pleger

**Affiliations:** ^1^Max Planck Institute for Human Cognitive and Brain SciencesLeipzig, Germany; ^2^Integrated Research and Treatment Center (IFB) Adiposity DiseasesLeipzig, Germany; ^3^Department of Internal Medicine Clinic for Endocrinology and Nephrology, University Hospital LeipzigLeipzig, Germany; ^4^Clinic for Cognitive Neurology, University Hospital LeipzigLeipzig, Germany

**Keywords:** brain plasticity, obesity, magnetic resonance imaging, physical fitness, exercise, brain derived neurotrophic factor, BDNF, brain metabolism

## Abstract

Previous cross-sectional studies on body-weight-related alterations in brain structure revealed profound changes in the gray matter (GM) and white matter (WM) that resemble findings obtained from individuals with advancing age. This suggests that obesity may lead to structural brain changes that are comparable with brain aging. Here, we asked whether weight-loss-dependent improved metabolic and neurotrophic functioning parallels the reversal of obesity-related alterations in brain structure. To this end we applied magnetic resonance imaging (MRI) together with voxel-based morphometry and diffusion-tensor imaging in overweight to obese individuals who participated in a fitness course with intensive physical training twice a week over a period of 3 months. After the fitness course, participants presented, with inter-individual heterogeneity, a reduced body mass index (BMI), reduced serum leptin concentrations, elevated high-density lipoprotein-cholesterol (HDL-C), and alterations of serum brain-derived neurotrophic factor (BDNF) concentrations suggesting changes of metabolic and neurotrophic function. Exercise-dependent changes in BMI and serum concentration of BDNF, leptin, and HDL-C were related to an increase in GM density in the left hippocampus, the insular cortex, and the left cerebellar lobule. We also observed exercise-dependent changes of diffusivity parameters in surrounding WM structures as well as in the corpus callosum. These findings suggest that weight-loss due to physical exercise in overweight to obese participants induces profound structural brain plasticity, not primarily of sensorimotor brain regions involved in physical exercise, but of regions previously reported to be structurally affected by an increased body weight and functionally implemented in gustation and cognitive processing.

## Introduction

Obesity is a major health burden and dramatically climbing incidence rates, especially in rapidly developing countries like China or India, lead to a demand to develop new therapeutic strategies as well as their assessment (Roman et al., 2015). However, key mechanisms of the central nervous system driving and sustaining overeating behavior are still not well understood.

During eating, the hypothalamus, as the brain’s homeostatic control site, receives homeostatic signals arising mostly from the gastrointestinal system, the pancreas, and fat depots (Hollmann et al., 2013). This homeostatic information merges with sensory information about the food, such as the food’s smell and taste, which seems to involve the anterior insular cortex together with the frontal operculum (i.e., primary gustatory cortex; Rolls, 2006). Guided by the sensory and homeostatic information, key regions of the reward circuit, such as the striatum and the medial orbitofrontal cortex, attribute rewarding values to the food (O’Doherty et al., 2001; Pelchat et al., 2004), whereas the hippocampus seems to be responsible for matching the information about the food to previous experiences (Weltens et al., 2014).

Based on these general assumptions on how hedonic, homeostatic, and memory-related brain sites commonly orchestrate everyday eating behavior, cross-sectional magnetic resonance imaging (MRI) studies started to investigate their structural alterations due to an increased body weight.

These studies either correlated the body mass index (BMI), the waist-to-hip ratio, or other measures of an increased body weight with MRI data of gray matter (GM) or white matter (WM), or compared these structural compartments between lean and obese individuals. The findings suggest weight-related altered GM in several brain areas, including regions implicated in the regulation of taste (i.e., anterior insula/frontal operculum and postcentral gyrus), reward processing (i.e., putamen), memory formation and retrieval (i.e., hippocampus), rational cognition (i.e., anterior cingulate cortex), relay (i.e., thalamus) and executive functioning (i.e., prefrontal lobe; Jagust et al., 2005; Pannacciulli et al., 2006; Raji et al., 2010; Walther et al., 2010). Other cross-sectional studies used measures of body fatness, like the blood’s leptin concentration or MRI-based abdominal fat scans, and found correlations with GM volume in the cerebellum, the inferior temporal gyrus, the frontal operculum, the postcentral gyrus, and the putamen (Pannacciulli et al., 2007; Raschpichler et al., 2013).

Studies that used diffusion-weighted MRI to investigate weight-related alterations of the water diffusivity in WM point to alterations across the entire corpus callosum (Mueller et al., 2011; Stanek et al., 2011), that share similarities with WM-related findings due to brain aging (Ota et al., 2006; Giorgio et al., 2010). What remained unclear was to which extent these changes in the corpus callosum’s water diffusivity related to the weight-related GM changes (Mueller et al., 2014).

In a group of lean to obese women, we found obesity-related structural alterations in the left putamen and right dorsolateral prefrontal cortex (DLPFC; Horstmann et al., 2011). Gray-matter alterations in the DLPFC were negatively correlated with the radial diffusivity in the entire corpus callosum. Within the genu of the corpus callosum, we found a positive correlation with the axial diffusivity. In posterior regions and inferior areas of the body of the corpus callosum, axial diffusivity correlated negatively with altered GM in left putamen (Mueller et al., 2014). These findings suggest that in women obesity-related alterations of GM in brain regions involved in executive control (i.e., DLPFC) and habit learning (i.e., putamen) might relate to alterations of associated WM fiber bundles within the corpus callosum.

Whether such GM and WM alterations represent cause or consequence of overeating behavior remains currently unanswered. This question is, however, important, regarding an improved understanding of central mechanisms driving and sustaining overeating behavior, but also for the identification of brain regions that may act as targets for brain-stimulation techniques or neurofeedback training. Whether such interventions underpin, accelerate, or even initiate weight-loss remains another area for future research.

On the search for weight-loss related structural brain alterations that parallel improved metabolic and enhanced neurotrophic functioning, we investigate the brain’s GM and WM of overweight to obese individuals who participated in a fitness course with intensive physical training twice a week over a period of 3 months.

In previous MRI studies, the most common changes in GM related to physical exercise and irrespective of changes in body weight, were found in the hippocampus (Thomas and Baker, 2013). These findings are in good agreement with animal research showing exercise-induced formation of new neurons in the hippocampus (Kobilo et al., 2011; Mustroph et al., 2012; Grégoire et al., 2014), and parallel improvements in spatial memory (van Praag, 2008), which together may point to a well-preserved evolutionary interplay. This effect is associated with the local upregulation of the brain-derived neurotrophic factor (BDNF) and seems to immediately respond to exercise-related changes in metabolic signals arising from the muscle fibers (for a review see van Praag et al., 2014).

In the present MRI study, we investigated the effects of regular physical exercise on the brain’s GM and WM in overweight to obese individuals, with a focus on alterations related to weight-loss and altered neurotrophic and metabolic blood markers. We hypothesized a significantly reduced BMI, as well as BDNF-related alterations in GM and WM of regions not only underpinning eating behavior, but also cognitive functioning. We expected these brain alterations to be associated with a reduction of metabolic markers of obesity, such as leptin, and an increase of high-density lipoprotein-cholesterol (HDL-C).

## Materials and Methods

### Data Acquisition

Sixteen young overweight and obese volunteers (nine female, age 27.2 ± 6.7 years, BMI 33.6 ± 5.9 kg/m^2^) participated in the experiments (Table 1). Exclusion criteria were depression (Beck’s Depression Inventory, cut-off value 18), a history of neuropsychiatric diseases, smoking, diabetes mellitus, hypertension, conditions, which are contraindications to MRI and visible abnormalities on a T1-weighted MRI scan. The study was carried out in accordance with the Declaration of Helsinki and approved by the Ethics Committee of the University of Leipzig. Participants gave their written informed consent prior to their participation.

**Table 1:** **List of participants and recorded serum parameters**.

ID	Sex	Age [years]	BMI (kg/m^2^)		Leptin (ng/mL)		HDL-C (mmol/L)		Chol (mmol/L)		BDNF (ng/mL)	
			Pre	Post	Pre	Post	Pre	Post	Pre	Post	Pre	Post
**A1**	m	23.0	41.4	39.2	23.5	21.6	0.89	1.12	3.48	3.59	11.66	10.39
**BC**	m	41.8	34.0	34.0	11.3	12.8	1.58	1.65	6.38	6.55	7.71	8.56
**BD**	f	22.6	30.5	28.6	34.0	31.5	0.85	0.88	4.53	4.44	11.28	12.67
**BF**	f	27.7	33.4	33.1	30.2	29.8	1.30	1.22	5.20	5.13	14.16	16.09
**D3**	m	36.4	30.7	31.0	9.2	10.5	1.36	1.41	6.35	6.33	10.17	9.48
**E2**	m	38.3	50.7	49.8	58.6	46.8	1.24	1.46	3.09	3.49	5.14	4.66
**K8**	m	22.9	28.7	25.9	5.3	4.9	1.56	1.69	4.32	4.41	9.45	11.63
**L5**	f	21.3	30.7	31.7	86.2	79.8	2.11	2.29	5.46	5.57	9.02	10.29
**LG**	m	24.6	34.7	34.7	22.0	23.5	0.92	1.05	5.08	5.10	6.41	8.67
**N2**	f	23.8	28.4	27.8	31.2	33.8	1.95	2.01	5.53	5.81	10.40	9.52
**P8**	m	35.2	34.5	33.3	15.0	15.4	0.92	0.99	6.28	6.13	13.35	15.34
**R9**	f	22.1	40.1	40.1	87.6	75.9	1.29	1.31	5.87	5.43	12.78	14.40
**SG**	f	20.6	30.1	29.7	58.8	60.2	1.80	1.74	4.81	4.72	7.58	9.64
**SE**	f	30.6	30.1	28.7	17.9	18.4	1.59	1.63	6.03	5.81	14.03	12.38
**SJ**	f	25.5	30.4	30.4	13.2	10.7	1.32	1.27	4.28	4.11	10.69	9.09
**S1**	f	26.0	29.2	28.7	20.9	16.9	1.62	1.85	3.78	4.03	12.34	14.66

*Abbreviations: BDNF, Brain-derived neurotropic factor; BMI, body-mass index; Chol, Cholesterol; HDL-C, high-density lipoprotein-cholesterol; post, post-exercise value; pre, pre-exercise value*.

Participants were examined in two sessions before and after a fitness course with intensive physical training over a period of 3 months. The primary goal was to lose weight, not to gain fitness. We also asked our participants to record their eating behavior over 7 days with food intake protocols. As per our instruction, there was no significant change in key parameters of food intake, such as daily total caloric (and macronutrient) intake, distribution of macronutrient intake or alcohol consumption. Based on the protocols, we assumed constant eating habits over the course of the 3 months.

The fitness course was funded by each participant’s health insurance. Individuals were enrolled in 60 min of supervised physical training twice a week as described previously (Blüher et al., 2007). In brief, each training session included 15 min of biking or running, 30 min of individualized strength training, and 15 min of warming up/cooling down periods. All participants completed a graded bicycle-ergometer test to volitional exhaustion and had maximal oxygen uptake measured with an automated open circuit gas analysis system at baseline. The highest oxygen uptake/minute reached was defined as the maximal oxygen uptake, and participants subsequently trained at their individual submaximal heart rate defined as 70–80% of the individual maximal heart rate during the bicycle-ergometer test.

Blood samples, as well as the body height and weight (to compute the BMI) were obtained in the fasting state, before and after the three-month training period. Blood was withdrawn into a serum vacutainer from a peripheral venous puncture in the elbow flexure. To separate the serum, blood was centrifuged at 4°C for 10 min at a relative centrifugal force of 3500 g. All baseline blood samples were collected between 8–10 am after an overnight fast (>8 h) and handled following standardized preanalytical protocols by two experienced technicians. Plasma insulin was measured with an enzyme immunometric assay for the IMMULITE automated analyzer (Diagnostic Products Corporation, Los Angeles, CA, USA). Besides leptin, HDL-cholesterol and BDNF (see Table 1), we also measured circulating insulin, proinsulin, albumin, creatinine, glucose, total protein, C-peptide, LDL-cholesterol, and total cholesterol.

In both sessions, MRI scanning was performed directly after blood withdrawal on a 3-Tesla TIM Trio system (Siemens, Erlangen, Germany) using a 12-element head matrix coil. Anatomical images were acquired using a T1-weighted three-dimensional magnetization-prepared rapid gradient echo (MP-RAGE) sequence with selective water excitation and linear phase encoding (Mugler and Brookeman, 1990). A sagittal slice orientation was used with the following imaging parameters: inversion time, TI = 650 ms; repetition time, TR = 1300 ms; echo time, TE = 3.5 ms; readout pulse flip angle, α = 10°; bandwidth = 190 Hz/pixel; image matrix = 256 × 240; field of view, FOV = 256 × 240 mm^2^; nominal spatial resolution = 1 × 1 × 1 mm^3^; 2 averages.

Diffusion-weighted images were acquired from 72 axial slices with 1.72-mm thickness (no gap) covering the entire brain with a twice-refocused spin echo echo-planar-imaging sequence (Reese et al., 2003): TE = 100 ms, TR = 12 s, α = 90°, bandwidth = 1345 Hz/pixel, FOV = 220 × 220 mm^2^, matrix: 128 × 128 acquired with 7/8 partial Fourier encoding and GRAPPA (acceleration factor 2). Diffusion-weighted data were acquired with 60 diffusion-encoding gradient directions and a b-value of 1000 s/mm^2^. In addition, seven volumes were recorded without diffusion weighting as anatomical reference for offline motion correction—one at the beginning of the scanning sequence and one after each block of 10 diffusion-weighted images. Random noise was reduced by averaging three acquisition cycles, resulting in a total acquisition time of about 45 min.

### Data Analysis

The exercise-related intra-individual change of serum markers was assessed using one-tailed paired *t*-tests with *p* < 0.05. Serum markers showing a significant change were subsequently used for investigating potential correlations with GM structure and diffusivity parameters.

T1-weighted MR images were processed using SPM8 (Wellcome Trust Centre for Neuroimaging, UCL, London, UK) and Matlab 7 (Mathworks, Sherborn, MA, USA). Images were transformed using the VBM8 toolbox that includes segmentation, bias-correction, and normalization using the Diffeomorphic Anatomical RegisTration using Exponentiated Lie algebra (DARTEL) technique (Ashburner, 2007) with a pre-defined tissue probability map registered to the Montreal Neurological Institute (MNI) space. Modulation was performed to compensate for the effect of non-linear transformations. Finally, a Gaussian filter of 8 mm full width at half maximum (FWHM) was applied. In agreement with general practice, we used the term *gray matter density* (GMD) to describe the normalized and modulated GM probability values.

Voxel-wise statistical analysis was performed using a paired *t*-test with pre- and post-measurement as the experimental factor. Parameters were estimated for all voxels with a minimum GMD of 10%. Differences between both sessions were detected with a voxel threshold of *p* < 0.001. To correct for multiple comparisons, significant clusters were obtained using family-wise error (FWE) correction, *p* < 0.05. Further, in order to investigate a relationship between GMD and the intra-individual alterations of serum markers, paired *t*-tests were accommodated to compare pre- with post-measurements including additional covariates implemented with SPM’s flexible factorial design. In the same way, we also checked for an exercise-related intra-individual relationship between BMI and GMD change.

Diffusion-weighted data were processed using the FMRIB Software Library (FSL; Smith et al., 2004). Motion correction was performed with rigid-body transformation (Jenkinson et al., 2002) using the non-diffusion-weighted reference images acquired during diffusion-weighted imaging. This processing step was combined with a global registration to the T1-weighted images. Then, diffusion-weighted images were skull-stripped using the individual T1-weighted image and finally co-registered to the standard MNI template. The gradient direction for each volume was corrected using the rotation parameters. The registered images were interpolated with an isotropic voxel resolution of 1 mm, and the three corresponding acquisitions and gradient directions were averaged. Finally, for each voxel, a diffusion tensor was fitted to the data, and diffusion parameters comprising apparent diffusion coefficient (ADC), fractional anisotropy (FA), axial diffusivity (λ_∥_), and radial diffusivity (λ_⊥_) were computed from the eigenvalues of the diffusion tensor.

Voxel-wise diffusivity parameters (ADC, FA, λ_∥_ and λ_⊥_) were statistically analyzed using TBSS (Smith et al., 2006) as implemented in FSL (Smith et al., 2004). For this purpose, a mean FA image was created and thinned to create a mean FA skeleton, which represents the centers of all tracts across participants. Next, each subject’s diffusivity parameters were projected onto this skeleton. Differences between diffusivity parameters of the pre- and post-measurement were detected with voxel-wise statistical randomization tests (Nichols and Holmes, 2002) using a paired design. Significant results were obtained using threshold-free cluster enhancement (TFCE) and correction for multiple comparisons at the *p* < 0.05 (corrected) level (Smith and Nichols, 2009). In addition to the comparison of pre- and post-measurements, the relationship between diffusivity parameters and serum markers were assessed using permutation tests with a flexible factorial design including a subject factor to account for intra-individual changes in the physiological parameters.

## Results

### Exercise-Induced Alterations in GMD and Diffusivity Parameters

**Figure 1:**
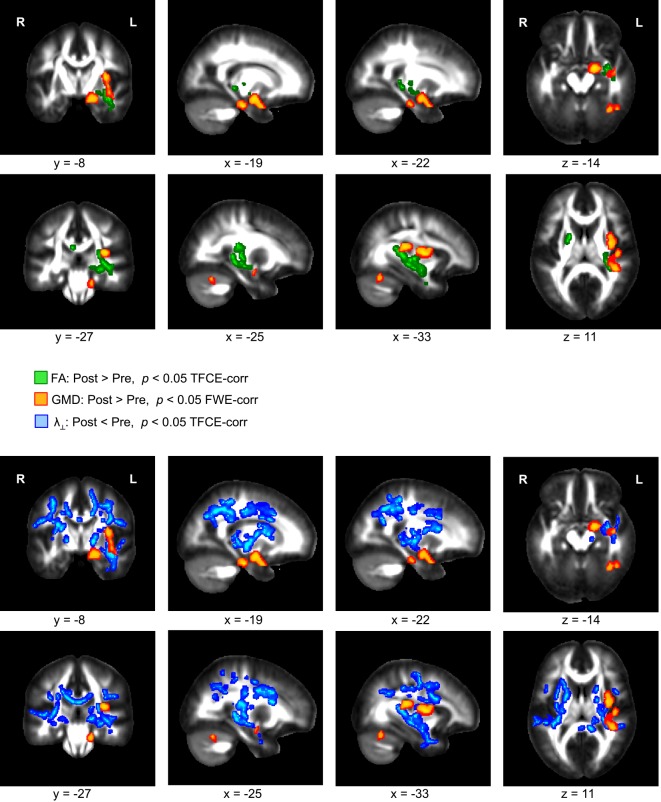
**Significant increase of gray matter density (GMD, color-coded in orange) and fractional anisotropy (FA, color-coded in green) in overweight and obese participants comparing images of two scanning sessions before and after a period of 3 months of physical exercise**. In addition, a decrease of radial diffusivity (λ_⊥_, color-coded in blue) was obtained when comparing diffusion weighted images of both scanning sessions.

A comparison of the T1-weighted MR images before and after the three-month exercise program yielded a significant increase of GMD in the left hippocampus, the left insular cortex, and the left cerebellum (Figure 1, orange). These GMD changes were accompanied by an FA increase in WM areas directly neighboring the left hippocampus and insular cortex (Figure 1, green). In addition to the FA increase, a reduction of λ_⊥_ was found in the same WM territories, and also in further regions including the entire corpus callosum (Figure 1, blue). We did not find any significant change of λ_∥_.

### Exercise-Induced Alterations in BMI, Leptin, HDL-C, and BDNF Serum Levels

**Figure 2:**
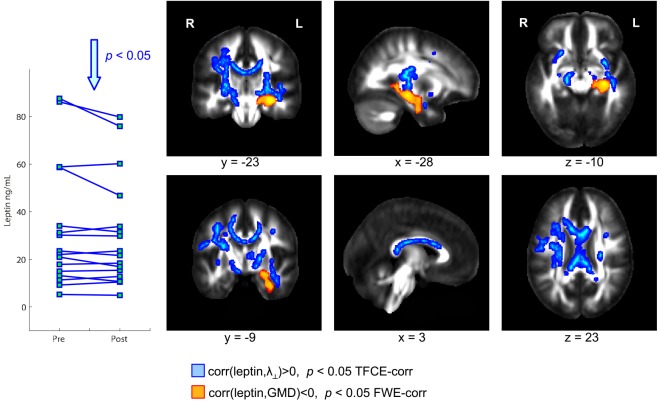
**Coronal, sagittal, and axial brain sections showing leptin-dependent correlations of gray and white matter (WM) structural parameters in overweight and obese participants**. Higher serum leptin levels were accompanied by a reduced gray matter density (GMD) in the hippocampus (color-coded in orange). Further, a significant positive correlation between leptin and radial diffusivity (λ_⊥_) was observed in wide-spread WM regions including the whole corpus callosum (color-coded in blue).

**Figure 3:**
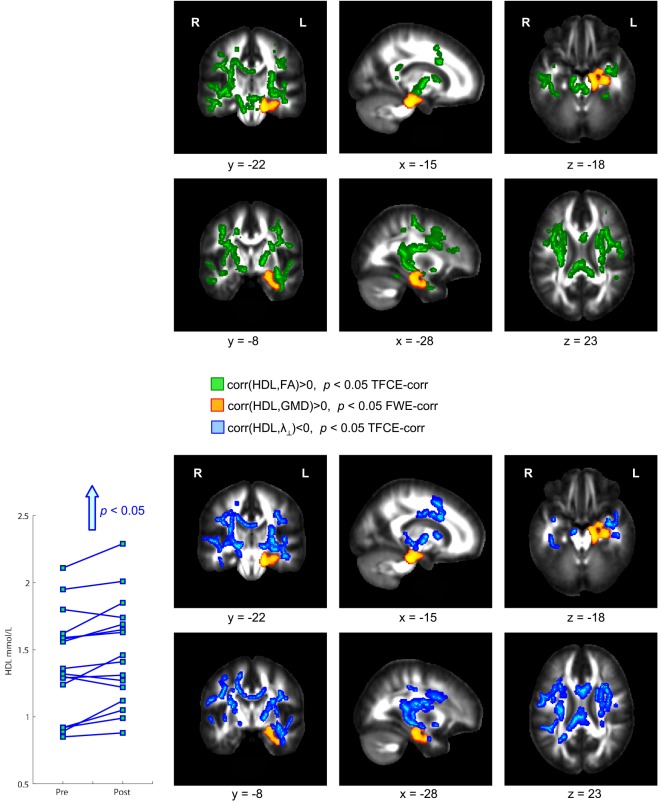
**Significant positive correlation between gray matter density (GMD) and high density lipoprotein-cholesterol (HDL-C) serum levels**. After the three-month fitness course, participants showed a significant HDL-C increase correlated with a GMD increase in hippocampal brain regions (color-coded in orange). This HDL-C increase was further accompanied with an increase of fractional anisotropy (FA, color-coded in green) and a decrease of radial diffusivity (λ_⊥_, color-coded in blue).

Physical exercise significantly reduced the BMI across the entire group of participants (paired *t*-test; *p* = 0.008). While we found a high degree of variability comparing the intra-individual serum leptin concentration before and after the three-months fitness course (Table 1), assessment of the whole group by a paired *t*-test yielded a significant decrease (*p* = 0.044; Figure 2; significance was driven by three participants showing a major leptin decrease). In contrast to such individual variability, we found HDL-C changes to be more consistent across participants. Over the course of the three-month fitness training, HDL-C significantly increased (*p* = 0.003; Figure 3). The BDNF values showed again a more heterogeneous picture: comparing the pre- and post-exercise BDNF values across the group, we found a significant BDNF increase with *p* = 0.041. However, only a subgroup of 10 participants showed this effect (“BDNF responders”), whereas the remaining six participants did not show an increase (“BDNF non-responders”). Interestingly, we did not observe a correlation between BDNF and leptin changes across participants, that is, we did not find a consistent leptin decrease in BDNF responders. All individual pre- and post-exercise measurements of BMI, leptin, HDL-C, and BDNF are listed in Table 1.

### Correlation of Exercise-Induced Changes of Serum Leptin, HDL-C, and BDNF with Hippocampal GMD and Diffusion Parameters in Adjacent WM Regions

**Figure 4:**
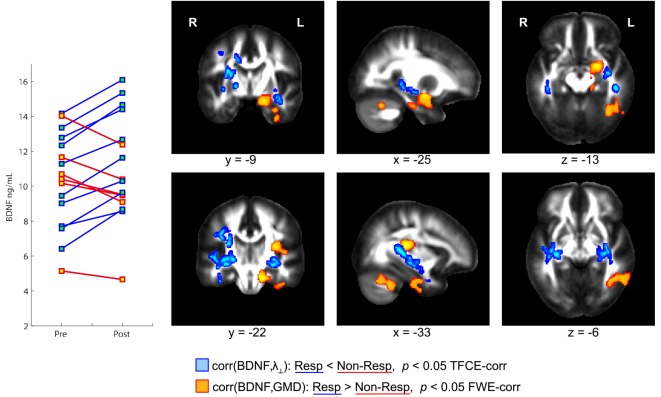
**Orthogonal brain sections showing structural brain differences between brain-derived neurotrophic factor (BDNF) responders and non-responders**. BDNF responders, that is, participants with a serum BDNF increase during the three-month fitness course, showed a significantly higher correlation between BDNF and gray matter density (GMD) compared to non-responders (see regions color-coded in orange). In responders, but not in non-responders, a BDNF increase is accompanied with a GMD increase in hippocampus, insula, and cerebellar regions within the left hemisphere. Responders and non-responders also differ with respect to the correlation between BDNF and radial diffusivity (λ_⊥_, color-coded in blue). In responders, but not in non-responders, a BDNF increase is accompanied with a decrease of λ_⊥_ in brain regions in the vicinity of the left hippocampus and left insular cortex.

The exercise-induced reduction in leptin levels (Figure 2) as well as the HDL-C (Figure 3) and BDNF increase (in BDNF responders as compared to BDNF non-responders, see Figure 4) were significantly correlated with increased GMD in the left hippocampus (colored in orange). In adjacent WM regions, we found a significantly reduced λ_⊥_ (colored in blue). In the vicinity of the hippocampus, we also observed an increased FA together with an increased HDL-C (Figure 3, top row, green).

Our findings suggest that the improvement in fat metabolism, as indicated by the reduced leptin and increased HDL-C levels, as well as the enhanced neurotrophic signaling, as indicated by increased BDNF levels, were paralleled by an increased GMD in the left hippocampus and an altered diffusivity in directly neighboring WM regions.

### Correlation of Exercise-Induced Changes of Serum Leptin and HDL-C with Diffusion Parameters in WM Regions Remote from the Hippocampus

We also found a positive correlation between reduction of leptin levels after exercise and a decrease in λ_⊥_ in the corpus callosum (Figure 2, blue). Also for the increased HDL-C levels we found correlations with the diffusivity of water molecules across the corpus callosum. Here, we observed that increased HDL-C levels positively correlated with increased FA values (Figure 3, top row, green) and negatively with the λ_⊥_ (Figure 3, bottom row, blue), suggesting an HDL-C- and leptin-related altered trans-callosal diffusivity of water molecules.

### Correlation of BDNF with GMD and Diffusivity Parameters

Those participants, who responded to physical exercise with an increase in BDNF levels, showed an enhanced (positive) correlation between BDNF levels and GMD in distinct brain regions as compared to participants responding with a decrease in BDNF levels. Such GMD changes were found in left hippocampus, and also in the left insular cortex as well as parts of the left inferior cerebellum (Figure 4, orange regions). Similarly, we found an enhanced (negative) correlation between BDNF levels and λ_⊥_ in BDNF responders compared to BDNF non-responders (see Figure 4, color-coded in blue). This observation was significant in the vicinity of the hippocampus, and also in WM regions adjacent to the left and right insular cortex.

### Correlation Between BMI and GMD

**Figure 5:**
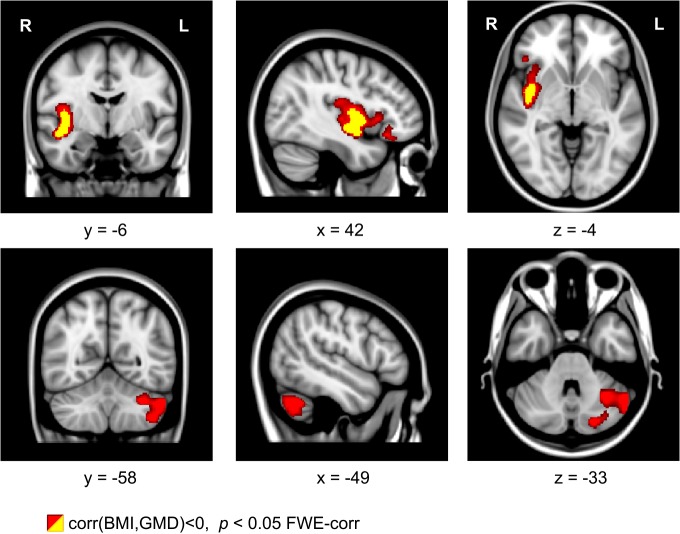
**Coronal, sagittal, and axial brain sections showing an intra-individual relationship between gray matter density (GMD) and body mass index (BMI) with physical exercise**. A decrease of BMI is related to an increase of GMD in the right insula and in left cerebellar regions. Clusters were generated using a voxel threshold of *p* < 0.001 (yellow) and *p* < 0.01 (red). Shown clusters are significant using family-wise error (FWE) correction with *p* < 0.05.

For the exercise-dependent reduction of BMI, we found a significant correlation with the increased GMD in the left cerebellum (see Figure 5, bottom row, color-coded in red). This correlation was located in the same cerebellar region as observed in the pre vs. post comparison (Figure 1). Using the SPM anatomy toolbox (Eickhoff et al., 2005) together with the probabilistic cerebellar atlas by Diedrichsen et al. (2009), the location was assigned to the left cerebellar lobule VIIa Crus I. Besides this correlation, we also found a correlation between the BMI reduction and GMD increase in the right insular cortex (see Figure 5, top row, color-coded in red and yellow).

## Discussion

Here, we show alterations in GM and WM structural parameters together with changes in metabolic and neurotrophic signaling in overweight to obese individuals, who participated in a three-month fitness course. After the training, participants presented a slightly, but significantly, reduced BMI, a reduced leptin serum concentration as well as an elevated HDL-C and BDNF serum concentration suggesting improved metabolic and enhanced neurotrophic functioning. The influence on participants’ BMI was overall small and inter-individually heterogeneous. Nevertheless, the effect seems comparable to other physical exercise studies. In a recent review paper, Swift and colleagues summarized recent studies that aimed to investigate the effect of physical exercise on body weight (Swift et al., 2014). They reported that exercise training, consistent with public health recommendations, promote up to modest weight loss (<2 kg), however the weight loss on an individual level was highly heterogeneous. Although our study sample is rather small when compared to the data reviewed by Swift and colleagues, the effect on body weight seem to match their findings (Swift et al., 2014). Exercise-dependent changes in the BMI, as well as changes in the serum concentration of BDNF, leptin, and HDL-C were, furthermore, related to an exercise-dependent increase in GMD in the left hippocampus, the left insular cortex, and the left inferior cerebellum as well as an increased FA, and a reduced λ_⊥_ in surrounding WM structures as well as the corpus callosum. The exercise-dependent reduction of the BMI, however, was negatively correlated with an increased GMD in the left inferior cerebellum and the right insular cortex, suggesting that the higher the decrease in BMI, the higher was the increase in cerebellar and insular GMD.

### Associations Between BDNF, Structural Brain Plasticity, and Metabolism

BDNF is a basic protein consisting of 252 amino acids. It is coded by the BDNF gene, which extends over 70 kb, located in humans on chromosome 11 (Maisonpierre et al., 1991; Binder and Scharfman, 2004; Pruunsild et al., 2007). Its protein levels are relatively high in the rat hippocampus and cortex, but BDNF is also measurable in the striatum and other structures, such as the olfactory bulbs (Conner et al., 1997; Ding et al., 2004). BDNF is well documented for its wide range of neurotrophic and neuroprotective functions, not only in the brain and spinal cord, but also in the periphery. Several lines of evidence converge on the notion that BDNF is involved in neuronal protection and survival, neurite expression, axonal and dendritic growth and remodeling, neuronal differentiation, and synaptic plasticity (Barde, 1994; Lindsay, 1994; Lewin, 1996; Alsina et al., 2001; Cotman and Berchtold, 2002).

Besides this relation to brain plasticity, BDNF also seems to moderate neuroendocrine and metabotrophic processes leading to lowered food intake as well as improved glucose metabolism and insulin sensitivity (Tsuchida et al., 2001; Nakagawa et al., 2002; Lebrun et al., 2006; Tsao et al., 2008; Yamanaka et al., 2008). Leptin, known for its key role in regulating appetite and energy metabolism, was found to interact with BDNF expression in the mouse hypothalamus, a brain structure well known for its involvement in regulating energy homeostasis (Komori et al., 2006). Additionally, when mice gained body weight, hippocampal BDNF levels declined, suggesting a direct link between energy metabolism and BDNF expression in the hypothalamus and in the hippocampus (Molteni et al., 2004).

There is also growing evidence that BDNF has a profound influence on metabolic processes in humans. Gray and colleagues reported a single case study of a subject with chromosomal inversion of a region encompassing the BDNF gene. Reduced serum BDNF levels were shown to be phenotypically associated with an impaired cognitive functioning and hyperactivity, but also with severe obesity (Gray et al., 2006), while increased serum BDNF levels were found in overweight to obese individuals after losing weight after a reduced-calorie diet (Araya et al., 2008). This study indicates that BDNF is not exclusively triggered by physical exercise since its serum levels also rise when consuming less calories. Taking together, BDNF seems not only essentially involved in structural brain plasticity, but also in central and peripheral molecular processes of energy metabolism and homeostasis (Knaepen et al., 2010).

In the present study, exercise-related changes in neurotrophic function (as indicated by elevated BDNF levels) were correlated with the exercise-dependent changes in parameters related to brain structure, especially in the left hippocampus. However, we did not find direct relationships between BDNF and leptin or HDL-C levels. Although the interpretation of negative findings is generally problematic (e.g., results may become significant upon increasing the sample size), this may suggest that a direct link between neurotrophic (i.e., BDNF) and metabolic signaling (i.e., leptin, HDL-C) is unlikely. However, levels of BDNF, leptin, and HDL-C were only assessed in the blood in our study (i.e., no information on hippocampal levels were available), which may also explain the differences between our results and previous observations in mice (Molteni et al., 2004; Komori et al., 2006).

### Physical Exercise, Metabolic Muscle-Brain Interactions and BDNF Expression

Physical exercise entails a wide repertoire of health benefits, such as increased erobic capacity, decreased adiposity, tighter regulation of glucose and insulin signaling, and increased cardiac output (Dishman et al., 2006; Anderson et al., 2010). Exercise is furthermore well documented to trigger mRNA expression and BDNF levels in the hippocampus with vast influences on energy metabolism as well as plastic brain processes (Neeper et al., 1995, 1996; Tong et al., 2001). The underlying biochemical mechanisms of this BDNF release have been intensively discussed. It is assumed that exercise generates low dose oxidative stress with positive effects on cell systems (Arumugam et al., 2006). A cascade of processes is initiated that are not only important for reparation, but also for remodeling and reshaping processes that probably intend to improve the system’s capacity for handling free oxygen radicals (Radak et al., 2008). This assumption may explain why basal BDNF levels are differentially regulated in individuals whose physiology has adapted to the metabolic demands of regular exercise (Whiteman et al., 2014).

In animal research, exercise was furthermore shown to induce skeletal muscle activation that immediately influences central nervous processing through transcriptional factors regulating muscle fiber contractile and metabolic genes (Wang et al., 2004). The peroxisome proliferator activated receptor (PPAR) regulates fast-twitch muscle fiber contraction and metabolism. PPAR overexpression was shown to increase oxidative muscle fiber number, and endurance when combined with exercise (Narkar et al., 2008). PPAR is controlled by the AMP-activated protein kinase (AMPK), a master metabolic regulator important for glucose homeostasis, appetite, and exercise physiology (Hardie, 2004). Kobilo et al. (2011, 2014) tested whether the AMPK agonist 5-aminoimidazole-4-carboxamide riboside (AICR) activates skeletal muscles and induces cognitive effects comparable with exercise. Their findings suggest that AICAR treatment can, in fact, enhance spatial learning and BDNF-related hippocampal neurogenesis in mice with and without exercising (Kobilo et al., 2011, 2014). These studies suggest that brain plasticity can be immediately triggered by exercise and other interventions activating AMPK and upregulating BDNF expression (van Praag et al., 2014). Whether such muscle-brain interactions are also responsible for the exercise-dependent GM and WM alterations in the present study remains, however, speculative.

Regarding the exercise related differences in BDNF release, we found that some of our participants responded with BDNF increases, whereas others presented BDNF levels lower than prior to the intervention. One possible explanation for these inter-individual discrepancies might be an inter-individually different genetic predisposition. For instance, Val66Met, a single nucleotide polymorphism (SNP) at nucleotide 196, encodes an amino acid substitution at codon 66 in the prodomain of the BDNF gene in humans (Egan et al., 2003; Chen et al., 2008). Twenty to thirty percent in the human population are carriers of this SNP (Shimizu et al., 2004; Casey et al., 2009). It was shown to be associated with a decreased BDNF response (Chen et al., 2008), probably triggering a reduced neurotrophic response to plasticity (Gratacòs et al., 2007; Casey et al., 2009). To which extend carriership of BDNF SNPs, such as Val66Met, might explain the different responses in our participants remains, however, speculative because genetic assessments were not available.

### Exercise-Related BDNF Increase and Cognitive Function

A growing literature supports the notion that physical fitness may not only reduce the risk of Alzheimer dementia or Parkinson’s disease, but also works against healthy age-related deficits in cognitive functioning (Yaffe et al., 2001; Kramer and Erickson, 2007; Anderson et al., 2010; Grazina and Massano, 2013; Intlekofer and Cotman, 2013). It was shown that individuals with higher levels of physical fitness presented better cognitive performance and less age-related loss of brain volumes as compared to their less fit peers (Yaffe et al., 2001; Weuve et al., 2004; Hillman et al., 2008; Erickson et al., 2011). Physical exercise seems to mostly benefit cognitive functions that are most vulnerable to decline with age or health status (Dishman et al., 2006), which might explain why effects observed in young, relatively healthy adults (as in our study) are less clear.

We did not assess cognitive or learning skills as the primary focus was on weight-loss-associated changes in metabolic and neurotrophic functioning that parallel changes in brain structure. Future studies on effects from physical exercise should account for parallel improvements in cognitive functioning, such as learning abilities and memory capacities, to better disentangle BDNF’s specific influence on brain structure, metabolism, and cognitive processes.

### Exercise-Induced Alterations in Diffusion Parameters in the Corpus Callosum

Besides hippocampus-associated WM changes, we also found that the corpus callosum presented an increased FA and a decreased λ_⊥_ after 3 months of exercise. Previous cross-sectional studies on obesity-associated changes in the brain’s WM revealed a positive correlation between an elevated BMI, a reduced FA and an increased λ_⊥_ in the corpus callosum (Mueller et al., 2011, 2014; Stanek et al., 2011). The regional pattern of changes in FA and λ_⊥_ showed similarities with diffusion tensor imaging results obtained from individuals at higher age (Ota et al., 2006), which might indicate an accelerated aging of WM in obese participants. Physical exercise, as shown in the present study, seems to reverse some of the alterations in diffusion parameters, suggesting that physical exercise might restore obesity-related regional WM alterations.

### Exercise-Induced Alterations of GMD in the Hippocampus and Cerebellum

Besides the altered water diffusivity in the corpus callosum and increased GMD in the hippocampus, we also found GMD increases in the left inferior cerebellum, an area that was previously reported to present reduced GMD with an increased visceral adipose tissue (Raschpichler et al., 2013). We note that the cerebellar GMD increases were not found in areas underpinning sensorimotor functioning, as one would expect due considering the physical exercise program, but in left cerebellar lobule VIIa Crus I previously reported to underpin cognitive processes (Stoodley and Schmahmann, 2010). Iglói and colleagues recently identified specific functional circuits linking left cerebellar lobule VIIa Crus I to medial parietal, medial prefrontal, and hippocampal cortices for non-motor aspects of navigation (Iglói et al., 2014). Considering the aforementioned hypothesis that physical exercise may improve learning and memory capacities due to structural changes affecting the hippocampus, the additional GMD increases in left cerebellar VIIa Crus I may point to an alternative explanation, namely that exercise parallels improvements in non-motor aspects of navigation. As the design of the present study does not permit further assessment of these hypotheses, future studies should investigate changes in learning abilities, memory capacities, or navigation skills that may occur in parallel to physical exercise.

### Exercise-Induced Increases in Gray Matter Density of the Insular Cortex and Altered Diffusivity of Adjacent White Matter Regions

Besides the exercise-induced alterations in the corpus callosum, the hippocampus, and the cerebellum, we also found alterations in GMD and diffusivity in the insula and adjacent WM regions, respectively. The insular cortex is a multisensory brain region, and its activation is thought to relate to the sense of ownership and agency (Farrer et al., 2003) or the subjective awareness and affective processing of bodily signals (Craig, 2002, 2004). The insula consists of an anterior and a posterior part divided by the central sulcus. In the present study GMD increased in the posterior part and the more posteriorly located parts of the anterior insular cortex in relation to exercise and BMI. The anterior insula is assumed to play a major role in viscerosensory (Oppenheimer et al., 1992) and interoceptive processing (for a review see Craig, 2009), whereas the posterior insula is thought to contain perceptual representations for bodily awareness (Karnath et al., 2005). Moreover, in junction with the caudal orbitofrontal cortex and the adjacent frontal operculum, the anterior insula is thought to constitute the primary gustatory cortex (Rolls, 2006). Apart from the pure sensory gustatory processing, the insula seems also involved in coding the rewarding and hedonic aspects of food items (Berthoud, 2011). Previous functional brain imaging studies accumulated convincing evidence that in obese as compared to lean individuals the insular cortex differently responds to food items as well as to states of hunger and satiety, suggesting abnormal gustatory processing in obese individuals (Frank et al., 2013). Furthermore, in task-free, so-called resting-state functional MRI studies, the insular cortex presented a decreased functional connectivity in obese individuals (Kullmann et al., 2013). The present findings of an increased insular GMD and increased FA in adjacent WM regions may point to an improved perception for food within a “rewired” or, in other words, better connected insular cortex. However, this interpretation remains speculative since we did not assess changes in gustatory abilities or food preferences in our participants.

### Limitations

Our study has several limitations that should be considered while interpreting the present findings: Firstly, the size of our sample is rather small (*n* = 16). Since the effect sizes within the brain in the context of our study are unknown, we cannot apply power analyses to evaluate the appropriate sample size (Guo et al., 2014). In the MRI data analyses, we always considered all 40,000 voxels throughout the entire brain, and we always corrected the results for all these voxels according to general practice using the FWE correction method for Voxel-based morphometry (VBM; Hayasaka and Nichols, 2003) and voxel-wise statistical randomization tests for the diffusion-weighted data (Nichols and Holmes, 2002). Nevertheless, future studies should consider larger groups to also investigate the specific influence of gender and age on the weight-loss related structural brain alterations. Another open question is why the brain effects reported in this manuscript were restricted only to the left hemisphere. This finding also needs to be confirmed by future brain imaging studies considering larger sample sizes. Secondly, we did not record the individual exercise success to relate the individual brain findings to specific trainings elements, such as the erobic or anerobic parts. Thirdly, we also did not record any changes in nutrition beyond the first 7 days of regular exercise. Although we instructed our participants not to change their eating habits over the course of the study, potential changes in nutrition may still account for some of the changes reported above. Fourthly, we did not assess any genetic BDNF polymorphisms that may have accounted for the observed variable BDNF responses to the exercise-related weight loss (responders vs. non-responders). Future studies with larger sample sizes should consider such genetic assessments to investigate how BDNF polymorphisms, such as the Val66Met SNP, may moderate the interindividual variability in BDNF responses and associated plastic brain alterations. Lastly, we did not include any cognitive tests, such as tests on spatial memory capacities (van Praag, 2008). Future studies should consider tests on attention, memory and executive function to better delineate how weight reduction due to exercise changes brain structure and related cognitive functioning in humans.

### Summary

Together, our findings suggest that physical exercise in overweight humans induces weight loss and improved metabolic and neutrophic functioning. At the same time, we observed indications of structural brain plasticity, not primarily in sensorimotor brain regions but in regions that are well known to be affected by obesity and involved in gustation and cognitive processing, such as the insular cortex, the hippocampus, and the left cerebellar regions. In face of the functional implementation of these regions, future studies are needed to investigate the mutual interplay between physical exercise, brain structure, eating behavior, gustation and cognitive skills.

## Data Availability Statement

The authors confirm that all data underlying the findings are fully available. Demographic details about the study’s participants and serum markers are available in Table 1. The T1- and diffusion weighted MRI data are deposited at the Max Planck Institute for Human Cognitive and Brain Sciences, Leipzig, Germany. Requests for this data may be sent to Dr. Roberto Cozatl (cozatl@cbs.mpg.de).

## Conflict of Interest Statement

The authors declare that the research was conducted in the absence of any commercial or financial relationships that could be construed as a potential conflict of interest.
